# Sevoflurane Postconditioning-Induced Anti-Inflammation via Inhibition of the Toll-Like Receptor-4/Nuclear Factor Kappa B Pathway Contributes to Neuroprotection against Transient Global Cerebral Ischemia in Rats

**DOI:** 10.3390/ijms18112347

**Published:** 2017-11-06

**Authors:** Jung-Won Hwang, Young-Tae Jeon, Young-Jin Lim, Hee-Pyoung Park

**Affiliations:** 1 Department of Anesthesiology and Pain Medicine, Seoul National University Bundang Hospital, Seoul National University College of Medicine, Seongnam 13620, Korea; jungwon@snubh.org (J.-W.H.); ytjeon@snubh.org (Y.-T.J.); 2 Department of Anesthesiology and Pain Medicine, Seoul National University Hospital, Seoul National University College of Medicine, Seoul 03080, Korea; limyjin@snu.ac.kr

**Keywords:** inflammation, sevoflurane postconditioning, neuroprotection

## Abstract

The anti-inflammatory actions of sevoflurane postconditioning are suggested as an important mechanism of sevoflurane postconditioning-induced neuroprotection against cerebral ischemia. Here, we determined whether the anti-inflammatory effects of sevoflurane postconditioning were mediated via inhibition of the toll-like receptor (TLR)-4/nuclear factor kappa B (NF-κB) pathway after global transient cerebral ischemia in rats. Forty-five rats were randomly assigned to five groups as follows: (1) control (10 min of ischemia, *n* = 10); (2) sevoflurane postconditioning (two periods of sevoflurane inhalation after ischemia for 10 min with a wash period of 10 min, *n* = 10); (3) resatorvid (intraperitoneal injection of a selective TLR-4 antagonist (3 mg/kg) 30 min before ischemia, *n* = 10); (4) sevoflurane postconditioning plus resatorvid (*n* = 10), and sham (*n* = 5). The numbers of necrotic and apoptotic cells in the hippocampal CA1 region, the expression levels of TLR-4, NF-κB, cleaved caspase-3, and tumor necrosis factor alpha (TNF-α) in the anterior part of each brain, and the serum levels of TNF-α, interleukin 6 (IL-6), and interleukin 1 beta (IL-1β) were assessed 1 day after ischemia. The necrotic cell counts and expression levels of TLR-4, NF-κB, caspase-3, and TNF-α in brain tissue as well as serum levels of pro-inflammatory cytokines (TNF-α, IL-6, and IL-1β) were significantly higher in the control group than in the other groups. Our findings suggest that the anti-inflammatory actions of sevoflurane postconditioning via inactivation of the TLR-4/NF-κB pathway and subsequent reduction in pro-inflammatory cytokine production, in part, contribute to sevoflurane postconditioning-induced neuroprotection after global transient cerebral ischemia in rats.

## 1. Introduction

Sevoflurane is an inhalation agent used for general anesthesia. Sevoflurane postconditioning has been highlighted in neuroprotection against cerebral ischemia/reperfusion (I/R) injury, as it exerts neuroprotective effects in various experimental models of cerebral injury [1,2,3,4,5,6]. Anti-inflammation is suggested as an important mechanism of inhalation anesthetic postconditioning-induced neuroprotection against cerebral I/R injury [3,7,8]. A previous study investigating the anti-inflammatory effects of sevoflurane postconditioning demonstrated that serum levels of pro-inflammatory cytokines (for example, tumor necrosis factor (TNF)-α and interleukin (IL)-1β) were considerably lower in rats treated with sevoflurane postconditioning during early reperfusion compared with those in rats with only transient global cerebral ischemia [3]. However, the exact mechanism underlying sevoflurane postconditioning-induced anti-inflammation after cerebral ischemia remains unclear. 

Toll-like receptors (TLRs), which respond to invading pathogens, pro-inflammatory cytokines, and environmental stresses, play a key role in the innate immune system. TLR signaling activates nuclear factor kappa B (NF-κB), which triggers the production of various pro-inflammatory cytokines such as TNF-α and IL-1β [9]. TLR-4, among the 13 known TLR subtypes, is activated in the brain after cerebral I/R injury, and its inhibition results in a significant reduction in cerebral I/R injury [9,10,11,12]. In a view of neuroprotection, the effects of pre- or postconditioning of inhalation agents on TLR-4-mediated anti-inflammation are not fully investigated, although a recent study demonstrated that isoflurane preconditioning provided neuroprotection in rats subjected to transient focal cerebral ischemia by suppressing microglial TLR-4 expression [13]. Moreover, no study has investigated the effect of sevoflurane postconditioning on TLR-4-related inflammatory responses that develop after global cerebral I/R injury.

In this study, we hypothesized that the anti-inflammatory effects of sevoflurane postconditioning via inhibition of the TLR-4/NF-κB pathway and the subsequent reduction in pro-inflammatory cytokine production would contribute to the neuroprotection against transient global cerebral I/R injury in rats.

## 2. Results

### 2.1. Hemodynamics

Both the mean arterial pressure (MAP) and regional cerebral blood flow (CBF), in subjects without cerebral I/R injury and treatment (Group SH), were higher than those in the other groups during the ischemic period (*p* < 0.01, Table S1). At 30 min following reperfusion, the MAP and regional CBF, in subjects with only cerebral I/R injury (Group C) and cerebral I/R injury plus resatorvid treatment (Group R), were significantly higher than those in subjects with cerebral I/R injury plus sevoflurane postconditioning treatment (Group S), subjects with cerebral I/R injury plus both sevoflurane postconditioning and resatorvid treatments (Group SR), and subjects in Group SH (all *p* < 0.01), and the pH was higher in Group SH compared with that in the other groups (in all groups, *p* < 0.05, except Group SR, where *p* < 0.01). After 30 min, the MAP in Groups S and SR recovered from sevoflurane postconditioning-induced hypotension, and the regional CBF in both groups increased as well.

### 2.2. Neurologic Deficit Score

The neurologic deficit score measured 24 h after ischemia was significantly higher in Group C than those in Groups S, R, SR, and SH (16.0 (15.5–19.3) vs. 9.0 (6.0–13.0), 9.0 (6.0–11.8), 10.0 (8.3–12.5), and 4.0 (2.0–4.5), respectively; all *p* < 0.01). Moreover, the score was lower in Group SH compared with Groups S, R, and SR (all *p* < 0.01).

### 2.3. Histological Exams

**Figure 1:**
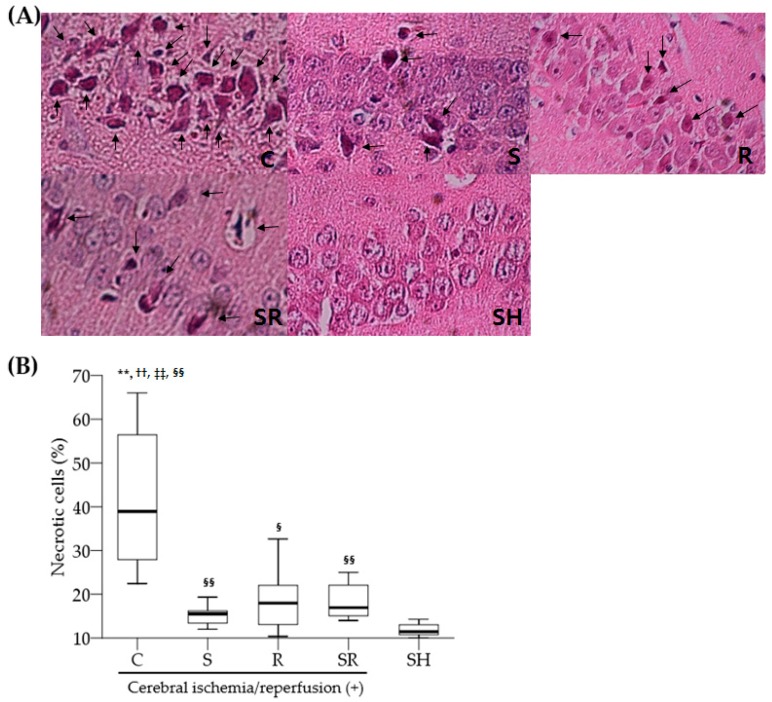
(**A**) Representative photomicrographs (×800) from a single rat with H&E staining in the hippocampal CA1 regions one day after transient global cerebral ischemia. Necrotic neurons (arrows) show karyolytic or pyknotic nuclei and cytoplasmic shrinkage; (**B**) The percentage of necrotic cells. Box plot indicates median, inter-quartile, and full ranges. C: control; S: sevoflurane postconditioning; R: resatorvid; SR: sevoflurane postconditioning + resatorvid; SH: sham. ** *p* < 0.01 for Group S; ^††^ *p* < 0.01 for Group R; ^‡‡^ *p* < 0.01 for Group SR; ^§§^ *p* < 0.01 for Group SH; ^§^ *p* < 0.05 for Group SH.

**Figure 2:**
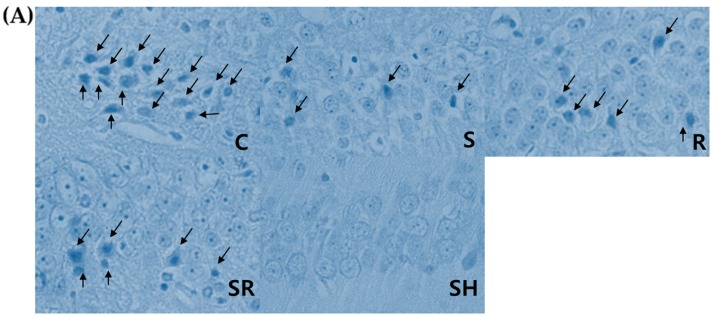

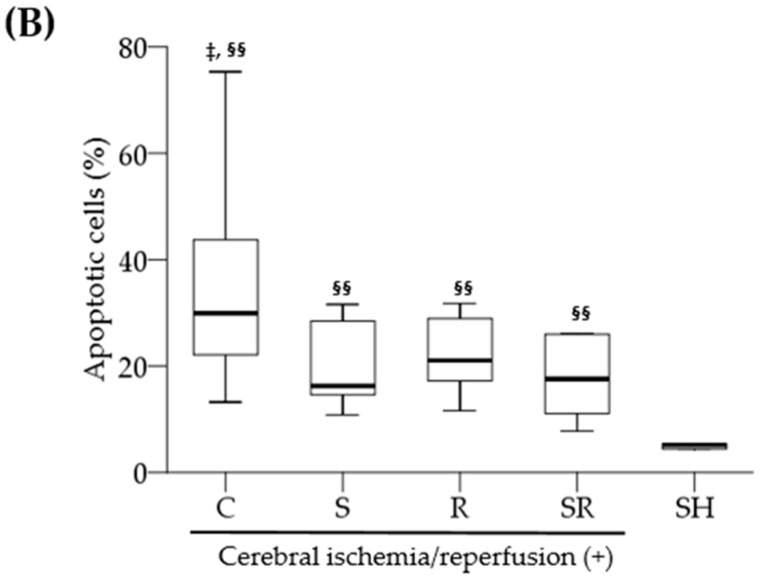
(**A**) Representative photomicrographs (×800) from a single rat with TUNEL staining in the hippocampal CA1 regions one day after transient global cerebral ischemia. Apoptotic cells (arrows) show a blue-stained apoptotic body. (**B**) The percentage of apoptotic cells. ^§§^ *p* < 0.01 for Group SH; ^‡^ *p* < 0.05 for Group SR.

A significant difference in the percentage of necrosis in the hippocampal CA1 on Post-Ischemic Day 1 was found among the five groups (*p* < 0.01; Figure 1A). The percentage of necrotic cells was significantly higher in Group C compared to Groups S, R, SR, and SH (all *p* < 0.01; Figure 1B), and was higher in Groups S, R, and SR compared to Group SH (*p* < 0.01 for Groups S and SR, *p* < 0.05 for Group R). Some apoptotic cells were observed in Groups C, S, R, and SR, whereas few were evident in Group SH (Figure 2A). The percentage of apoptotic cells in the hippocampal CA1 on Post-Ischemic Day 1 was higher in Groups C, S, R, and SR compared to Group SH (all *p* < 0.01; Figure 2B), and higher in Group C compared to Group SR (*p* < 0.05).

### 2.4. Western Blots

**Figure 3:**
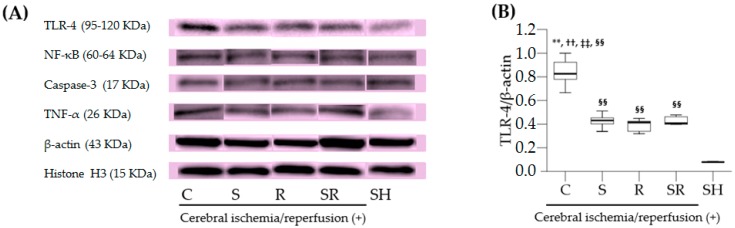

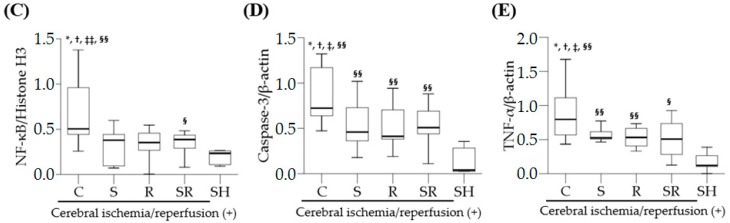
(**A**) Representative Western blot analysis of toll-like receptor 4 (TLR-4), nuclear factor kappa B (NF-κB), caspase-3, and tumor necrosis factor-alpha (TNF-α) one day after transient global cerebral ischemia. The relative expression levels of TLR-4 (**B**), NF-κB (**C**), cleaved caspase-3 (**D**), and TNF-α (**E**). β-actin and histone H3 serve as controls for cytoplasmic proteins (TLR-4, cleaved caspase-3, and TNF-α) and nuclear proteins (NF-κB), respectively. KDa: Kilo Dalton. ** *p* < 0.01 for Group S; ^††^ *p* < 0.01 for Group R; ^‡‡^ *p* < 0.01 for Group SR; ^§§^ *p* < 0.01 for Group SH. * *p* < 0.05 for Group S; ^†^ *p* < 0.05 for Group R; ^‡^ *p* < 0.05 for Group SR; ^§^ *p* < 0.05 for Group SH.

The relative expression levels of TLR-4, NF-κB, cleaved caspase-3, and TNF-α one day after ischemia are shown in Figure 3A. Cytoplasmic TLR-4 expression was higher in Group C than in Groups S, R, SR, and SH (all *p* < 0.01; Figure 3B), and higher in Groups S, R, and SR than in Group SH (all *p* < 0.01). The NF-κB expression in the nucleus was higher in Group C than in Groups S, R, SR, and SH (*p* < 0.05 for Groups S and R, and *p* < 0.01 for Group SR and SH; Figure 3C). Moreover, the cytoplasmic expression of caspase-3 was higher in Group C than in Groups S, R, SR, and SH (*p* < 0.05 for Groups S, R, and SR, and *p* < 0.01 for Group SH; Figure 3D). Lastly, TNF-α expression was higher in Group C than in Groups S, R, SR, and SH (*p* < 0.05 for Groups S, R, and SR, and *p* < 0.01 for Group SH; Figure 3E).

### 2.5. Serum Levels of Pro-Inflammatory Cytokines

**Figure 4:**
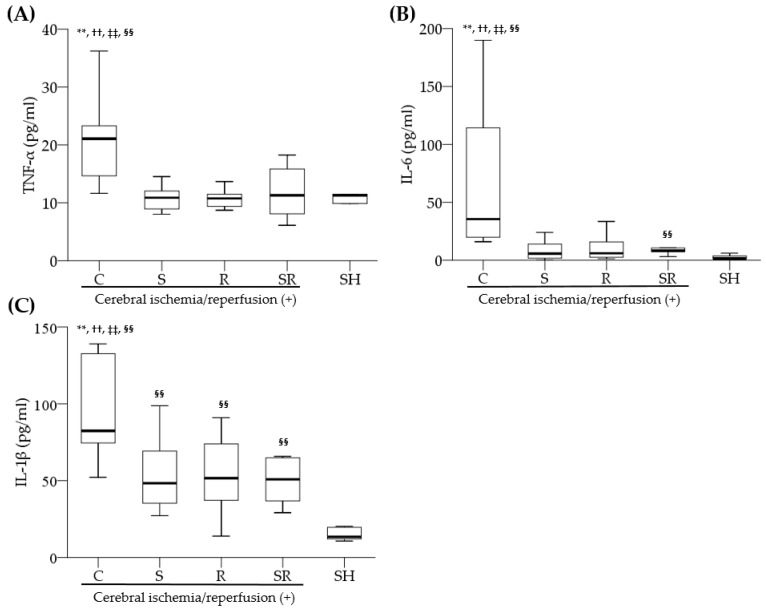
Serum levels of (**A**) tumor necrosis factor-alpha (TNF-α), (**B**) interleukin-6 (IL-6), and (**C**) interleukin1 beta (IL-1β) measured 24 h following transient global cerebral ischemia and assayed by ELISA. ** *p* < 0.01 for Group S; ^††^ *p* < 0.01 for Group R; ^‡‡^ *p* < 0.01 for Group SR; ^§§^ *p* < 0.01 for Group SH.

The serum levels of TNF-α (Figure 4A), IL-6 (Figure 4B), and IL-1β (Figure 4C) measured 24 h following ischemia were significantly higher in Group C than in Groups S, R, SR, and SH (all *p* < 0.01).

## 3. Discussion

This study shows that sevoflurane postconditioning decreased inflammation by inhibiting the TLR-4/NF-κB pathway and subsequent TNF-α expression in brain tissue after transient global cerebral I/R injury in rats. In addition, sevoflurane postconditioning reduced the expression of caspase-3 in the brain as well as serum levels of pro-inflammatory cytokines in peripheral blood. 

Inflammation is a crucial mechanism involved in the pathophysiology of cerebral I/R injury. Inflammation occurs in the early phase of cerebral I/R injury and can further aggravate neuronal injury [14]. Complex interlinked molecular and cellular mechanisms such as infiltrated leukocytes, oxidative stress, pro-inflammatory cytokines, and activated cyclooxygenase-2 are known to be involved in post-ischemic inflammation leading to cell necrosis and apoptosis [15]. Specifically, increased pro-inflammatory cytokine production via activation of the TLR-4/NF-κB pathway plays a pivotal role in post-ischemic inflammation. Numerous previous studies have reported that cerebral I/R injury dramatically increased the expression of TLR-4 in glial cells, microglia, neurons, and astrocytes, and that TLR-4 signaling increased the production of pro-inflammatory cytokines such as TNF-α, IL-6, and IL-1β in the brain through NF-κB activation [10,16,17,18]. Similarly, this study shows that the expression levels of TLR-4, NF-κB, and TNF-α were significantly higher in rats with cerebral I/R injury than in those without cerebral I/R injury, indicating that inflammation via activation of the TLR-4/NF-κB pathway followed by the overproduction of pro-inflammatory cytokines plays a crucial role in the pathophysiology of global cerebral I/R injury.

Sevoflurane postconditioning is known to provide neuroprotection against cerebral I/R injury [1,2,3,4,5,6]. The anti-inflammatory actions of sevoflurane postconditioning play an important role in sevoflurane postconditioning-induced neuroprotection. A previous study conducted by Seo et al. [3] demonstrated that sevoflurane postconditioning significantly reduced necrotic and apoptotic cells, as well as serum levels of pro-inflammatory cytokines, after transient global cerebral I/R injury in rats. However, in their study, the expression of pro-inflammatory cytokines was not investigated in the brain. Moreover, there was no explanation of the roles of TLR-4 and NF-κB, which are important upstream mediators of pro-inflammatory cytokines. In this study, sevoflurane postconditioning reduced the expression levels of TLR-4, NF-κB, and TNF-α in the brain; the extent of this decrease was similar to that shown with resatorvid treatment. Similar to this study, sevoflurane postconditioning-induced anti-inflammation has been reported to protect organs and cells from various injuries. A recent study demonstrated that sevoflurane postconditioning reduced myocardial damage significantly after myocardial I/R injury via inhibition of the TLR-4/NF-κB pathway and pro-inflammatory cytokine production [19]. Another study showed that sevoflurane postconditioning yielded cell protection by dramatically reducing the expression levels of TLR-4 and the pro-inflammatory cytokines TNF-α and IL-6 in human umbilical vein endothelial cells exposed to lipopolysaccharide [20].

Over the past few decades, the TLR-4/NF-κB pathway has gained increasing attention because its inhibition results in neuroprotection against cerebral I/R injury. Resatorvid, a selective TLR-4 antagonist, has been reported to exert a neuroprotective effect by decreasing inflammation in various experimental models of cerebral injury such as traumatic brain injury, cerebral I/R injury, and intracerebral hemorrhage-induced brain injury [11,21,22]. Various agents that exert TLR-4 suppressing effects, including dexmedetomidine, progesterone, and oxymatrine, have also been shown to provide neuroprotection against transient global cerebral I/R injury or cerebral hemorrhagic injury by reducing TLR-4 signaling-mediated inflammation [12,23,24]. In accordance with the aforementioned studies, this study confirms that resatorvid treatment had beneficial neuroprotective effects after transient global cerebral I/R injury through resatorvid-induced anti-inflammation.

Pro-inflammatory cytokines in serum and brain tissue after cerebral I/R injury can communicate with each other due to increased blood–brain-barrier (BBB) permeability. Pro-inflammatory cytokines in serum affect TLR-4/NF-κB activity in the brain indirectly; they enter injured brain sites through the damaged BBB. They strengthen TLR-4/NF-κB activity since they act as potent ligands of TLR-4. Moreover, serum levels of pro-inflammatory cytokines may reflect the extent of inflammation in the brain after cerebral I/R injury. In other words, the damage to membranes of neuronal and glial cells allow pro-inflammatory cytokines to move to the extracellular space. These cytokines enter the blood through the damaged BBB. Indeed, a previous study measuring the levels of pro-inflammatory cytokines in the brain and serum after cerebral ischemic and hypoxic injury showed that pro-inflammatory cytokines were significantly increased at both sites, and serum levels of pro-inflammatory cytokines were correlated with the severity of brain damage [25]. In this study, sevoflurane postconditioning markedly decreased TNF-α, IL-6, and IL-1β levels in peripheral blood as well as the expression of TNF-α in the brain after cerebral I/R injury, suggesting that sevoflurane postconditioning has anti-inflammatory actions by directly inhibiting the TLR-4/NF-κB pathway in damaged brain tissue and by indirectly reducing serum levels of pro-inflammatory cytokines and thereby suppressing TLR-4/NF-κB activity in the brain.

Inflammation is associated with apoptosis, which plays a key role in increased neuronal cell death after cerebral I/R injury. A recent experimental study in rats with diabetes and middle cerebral artery occlusion demonstrated that inhibition of the TLR4 signaling pathway significantly decreased neuronal cell apoptosis [26]. Caspase-3, which is a final and key enzyme of the intrinsic apoptotic cascade for neuronal apoptosis, was specifically involved [27]. The expression of caspase-3 is reported to be markedly increased after transient global cerebral I/R injury [5,12]. In the present study, sevoflurane postconditioning significantly diminished the expression of caspase-3 on Post-Ischemic Day 1. Such findings suggest that the anti-apoptotic effects of sevoflurane postconditioning via reduced caspases-3 expression are also responsible for sevoflurane postconditioning-induced neuroprotection.

In this study, we expected a synergistic neuroprotective effect in sevoflurane postconditioning plus resatorvid group because sevoflurane postconditioning is known to exert neuroprotection via other mechanisms as well as TLR-4-mediated anti-inflammation. Indeed, previous experimental studies showed that sevoflurane postconditioning provided neuroprotection by strengthening antioxidative effects via activation of nuclear factor erythroid 2-related factor and mitochondrial adenosine triphosphate potassium channel, and by weakening excitotoxicity via suppression of glutamate and *N*-methyl-d-aspartate receptor [5,28,29]. However, our results, in contrast to our expectation, indicated that combination of sevoflurane postconditioning and resatorvid showed no additive neuroprotective effects compared with either sevoflurane postconditioning or resatorvid alone. A possible explanation for such results is that the neuroprotective effects of either sevoflurane postconditioning or resatorvid are at a maximum, leaving little room for additivity. The combined use of their lower doses might allow additional neuroprotection.

Interestingly, this study showed that both sevoflurane postconditioning and resatorvid decreased the expression of TLR-4 in the brain after transient global cerebral I/R injury to the same extent. However, we did not explain the exact molecular mechanisms by which sevoflurane postconditioning and resatorvid decrease the expression of TLR-4 in this study. Resatorvid binds to TLR-4 selectively and interferes with the interactions between TLR-4 with adaptor molecules such as myeloid differentiation primary response gene 88, resulting in the inhibition of TLR-4 signal transduction and its downstream signaling events [30]. Therefore, resatorvid administration can exert neuroprotection in various experimental models of cerebral injury by functionally inhibiting TLR-4 and its downstream signal transduction, but not quantitatively suppressing the expression of TLR-4. Nonetheless, a recent study in a rat model of traumatic brain injury demonstrated that resatorvid administration prior to the injury significantly reduced the expression of TLR-4 at 12, 24, and 48 h following traumatic brain injury [21]. A possible explanation for such a decrease in the expression of TLR-4 shown in rats treated with sevoflurane postconditioning and resatorvid is that anti-inflammatory effects induced by both agents may affect the expression of TLR-4. In other words, we believe that they initially provide neuroprotective effects by decreasing neuroinflammation via inhibition of the TLR-4/NF-κB pathway and subsequent reduced production of pro-inflammatory cytokines, and thereafter such neuroprotection suppresses a further increase in TLR-4 expression induced by transient global cerebral I/R injury. Further studies are needed to determine whether TLR-4 suppression is a mediator or end-product of neuroprotection induced by sevoflurane postconditioning and resatorvid. 

In this study, there was a relatively large variation in the data shown in histopathologic, Western blot, and enzyme-linked immunosorbent assay (ELISA) examinations in the control group. A major disadvantage of global cerebral ischemia model using arterial hypotension plus bilateral common carotid artery (CCA) occlusions is that there is some inter-animal inconsistency with respect to both CBF decrement and pathologic outcome [31]. A possible explanation for such a finding is that the extent of global cerebral ischemia may differ in experimental rats. Although regional CBF was monitored in the unilateral cerebral hemisphere and the experimental model of global cerebral ischemia has been well established, there is a possibility that the amount of collateral CBF via the vertebral artery may be different between experimental rats and between the right and left cerebral hemisphere even in the same rat because both vertebral arteries were not occluded in this study. Indeed, a few experimental rats showed a remarkable difference in the number of apoptotic and necrotic cells between the right and left hippocampal CA1 area, suggesting that a difference in posterior collateral circulation via the vertebral artery might in part attribute to non-homogenous experimental results shown in the control group. 

With respect to hemodynamic changes, a significant reduction in MAP was observed in subjects with sevoflurane postconditioning in the early period of reperfusion due to vasodilatory effect of sevoflurane inhalation. Although sevoflurane results in hypotension, cardiac output is generally maintained via an increase in heart rate. Moreover, after sevoflurane postconditioning of 10 min, the washout period of 10 min was provided. Therefore, we think that a possibility of cerebral hypoperfusion resulting from temporary hypotension is low during sevoflurane postconditioning with short duration.

This study has some limitations. First, we did not determine the dose-dependent beneficial neuroprotective effects of sevoflurane postconditioning as only a fixed dose of sevoflurane (2.5 vol %) was used in this study. Second, we did not perform an immunofluorescence assay for glial cells, especially microglia, which is mainly responsible for neuroinflammation. Thus, we did not know the extent of activation of glial cells in the brain. In addition, histopathological data on brain glial cells, which are needed to demonstrate the potential communication between the peripheral and central immune system, were not provided. Next, the long-term positive effects of sevoflurane postconditioning on suppression of the TLR-4/NF-κB pathway were not investigated. Activation of the inflammatory cascade can take place for several days after cerebral I/R injury [14]. Finally, among the various TLRs, only TLR-4 was investigated. However, previous studies have reported that TLR-2 plays a crucial role in the development of neuronal damage after cerebral I/R injury [32,33]. Thus, additional studies exploring the effects of sevoflurane postconditioning on other types of TLRs after cerebral I/R injury are needed.

## 4. Materials and Methods

This experimental study was performed with approval from the Seoul National University Hospital Institutional Animal Care and Use Committee (IACUC No. 2016-0103, 01/02/2016). All animal care and experiments followed the Guide for the Care and Use of Laboratory Animals publicized by the National Institutes of Health. Male Sprague–Dawley rats aged 10–16 weeks and weighing 340–380 g each were housed under a 12 h day/night cycle at 20 °C. The rats were fasted for 12–16 h before the experiments and were provided with water ad libitum.

The rats were anesthetized with intraperitoneal (i.p.) zoletil (20 mg/kg) and xylazine (5 mg/kg) injections. After tracheal intubation, the rats received mechanical ventilation with 50% oxygen. Additional zoletil (10 mg/kg, i.p.) and xylazine (5 mg/kg, i.p.) were given when corneal reflexes and responses to tail pinching were observed. The femoral artery was catheterized for continuous arterial blood pressure monitoring and blood sampling. A rectal probe was inserted to measure and maintain the core body temperature at 37 °C using a heating/cooling board. A subcutaneous thermistor (TCAT-2 Temperature Controller; Harvard Apparatus, Holliston, MA, USA) was also inserted below the right temporalis muscle to monitor and maintain brain temperature at approximately 37.5 °C using an infrared lamp.

Forty-five rats were randomly assigned to one of five groups (Figure S1) as follows: (1) the control group (Group C, *n* = 10), which underwent transient global cerebral ischemia for 10 min; (2) the sevoflurane postconditioning group (Group S, *n* = 10), which received two periods of inhalation of 2.5 vol % sevoflurane for 10 min followed by a washout period of 10 min after ischemia [1]; (3) the resatorvid group (Group R, *n* = 10), which received resatorvid, a selective TLR-4 antagonist, at 3 mg/kg i.p. 30 min before ischemia [12]; (4) the sevoflurane postconditioning plus resatorvid group (Group SR, *n* = 10), which received resatorvid (3 mg/kg) 30 min before ischemia and sevoflurane postconditioning after ischemia; and (5) the sham group (Group SH, *n* = 5), in which both CCAs and the right jugular vein were surgically exposed. However, they received neither transient global cerebral ischemia nor any treatment.

Transient global cerebral ischemia was achieved as described previously [1]. Briefly, ischemia was induced via a combination of hemorrhage-induced arterial hypotension and bilateral CCA ligation. A laser Doppler flowmeter (moorVMS-LDF2; Moor Instruments, Axminster, UK) was used to monitor CBF during the procedure. The sensor of the monitor was placed and fixed with bone cement at the point (about 5 mm lateral to the midline and 2 mm posterior to bregma) on the left- or right-sided skull surface. For blood extraction and re-infusion, the right jugular vein was catheterized. After heparinization (50 U), blood was quickly drawn until the MAP decreased to 25–30 mmHg and the regional CBF was reduced to 50% of baseline. Both CCAs were then clamped with vascular clips for 10 min and the MAP was maintained at 25–30 mmHg during ischemia. The clips were carefully removed for reperfusion and the withdrawn blood was slowly re-infused. After the completion of surgical procedure, 0.5% bupivacaine was injected around the incision sites. 

Blood sampling for arterial blood gas analysis and hemoglobin and glucose measurement was performed 10 min before ischemia, during ischemia, and 30 min after reperfusion.

One day following ischemia, neurological outcome was evaluated by an investigator blinded to the animal groups with the modified neurological deficit scoring system described previously by Katz et al. [34] (Table S2). Rats were subsequently anesthetized with 20 mg/kg of zoletil (i.p.) and decapitated. After removing the brain, we transversely divided the brain into two parts using a rat brain slicer. The anterior part of each brain including a part of the hippocampus was stored in liquid nitrogen tank at −80 °C for Western blot analysis, while the posterior part was placed in buffered 10% formalin for microscopic histological examination. Paraffin wax-embedded brain tissues were serially cut with a 5 µm thickness for hematoxylin and eosin (H&E) and terminal deoxynucleotidyl transferase dUTP nick end-labeling (TUNEL) staining. An apoptosis detection kit (S7100, Millipore Corp., Billerica, MA, USA) was used for TUNEL staining. An investigator blinded to group assignment evaluated the total number of cells and the number of necrotic or apoptotic cells in the hippocampal CA1 region using a light microscope [1]. Necrotic neurons were identified by karyolytic or pyknotic nuclei and cytoplasmic shrinkage. TUNEL-positive cells with blue-stained apoptotic bodies were considered to be apoptotic cells. Six optical fields (left, three; right, three) of the hippocampal CA1 area were examined with the light microscope under high-power magnification (×400); the percentage of necrotic or apoptotic cells was calculated as the ratio of the number of necrotic or apoptotic cells to the total number of cells in each field. 

Western blotting for TLR-4, NF-κB, cleaved caspase-3, and TNF-α was performed using anti-TLR-4, anti-NF-κB p65, anti-cleaved caspase-3, and anti-TNF-α antibodies (Cell Signaling Technology, Beverly, MA, USA). The transversely excised brain tissues were cut into small pieces of 20–100 mg. Cytoplasmic and nuclear proteins were extracted using the NE-PER^®^ Cytoplasmic and Nuclear Extraction Kit (Thermo Scientific, Waltham, MA, USA) according to the manufacturer’s guidelines. Densitometry was used to quantify the band intensities. β-actin and histone H3 served as controls for cytoplasmic proteins (TLR-4, cleaved caspase-3, and TNF-α) and nuclear proteins (NF-κB), respectively.

Blood was extracted from the retro-orbital plexus 24 h following ischemia. The blood was centrifuged at 3000× *g* for 20 min. The serum levels of TNF-α, IL-6, and IL-1β were then assayed by ELISA kits (Sigma-Aldrich, St. Louis, MO, USA).

### Statistical Analysis

The primary endpoint was the expression level of TLR-4. The secondary endpoints were the expression levels of NF-κB, cleaved caspase-3, and TNF-α in the brain, the serum levels of TNF-α, IL-6, and IL-1β, and the percentages of necrotic and apoptotic cells in the hippocampal CA1 region. In a previous study, the expression ratio of TLR-4/β-actin was approximately 0.58 ± 0.20 following cerebral I/R injury in rats [15]. We assumed that a 50% decrease in the expression ratio of TLR-4/β-actin in Group S would be considered significant. Considering a type I error of 0.05 and a power of 80%, 10 rats in each group were needed. 

The expression levels of TLR-4, NF-κB, cleaved caspase-3, and TNF-α, the serum levels of TNF-α, IL-6, and IL-1β, the neurologic deficit score, and the percentages of necrotic and apoptotic cells in the hippocampal CA1 region were compared using the Kruskal–Wallis test followed by the Mann–Whitney U test. Physiological variables were compared using a repeated-measures analysis of variance (ANOVA), and the levels at each time point were analyzed using the Kruskal–Wallis test followed by the Mann–Whitney U test. A *p*-value <0.05 was considered to indicate statistical significance. All values were expressed as medians with interquartile ranges (Q1–Q3). SPSS software (version 22.0; IBM Corp., Armonk, NY, USA) was used for all statistical analyses. 

## 5. Conclusions

This study demonstrated that sevoflurane postconditioning provided neuroprotection against transient global cerebral I/R injury in rats by decreasing inflammation through inhibition of the TLR-4/NF-κB pathway and subsequent TNF-α expression in the brain, and by a reduction in pro-inflammatory cytokine production in the periphery. These findings suggest that the anti-inflammatory actions of sevoflurane postconditioning at dual sites partly contribute to sevoflurane postconditioning-induced neuroprotection after cerebral I/R injury.

## Supplementary Materials

Supplementary materials can be found at www.mdpi.com/1422-0067/18/11/2347/s1.

## Abbreviations

I/R	ischemia/reperfusion
TNF	tumor necrosis factor
IL	interleukin
TLR	toll-like receptors
NF-κB	nuclear factor kappa B
CCA	common carotid artery
CBF	cerebral blood flow
MAP	mean arterial pressure
H&E	hematoxylin and eosin
TUNEL	terminal deoxynucleotidyl transferase dUTP nick end-labeling
