# Supplementary material for: Sevoflurane Postconditioning-Induced Anti-Inflammation via Inhibition of the Toll-Like Receptor-4/Nuclear Factor Kappa B Pathway Contributes to Neuroprotection against Transient Global Cerebral Ischemia in Rats
Source: Int J Mol Sci. 2017 Nov 6;18(11):2347. doi: 10.3390/ijms18112347 (PMC5713316; doi:10.3390/ijms18112347)
Supplement: Supplementary file 1 [file ijms-18-02347-s001.zip › supplementary table 2.docx]

Table 2 Modified neurologic deficit scoring system

|  | Score |
| --- | --- |
| General behavioral deficit (worst = 10 points) |  |
| Consciousness  Attempt to explore spontaneously  No attempt to explore spontaneously (comatose) | 0  5 |
| Respiration  Normal  Abnormal | 0  5 |
| Cranial nerve reflexes (worst = 20 points) |  |
| Olfactory (sniffing food)  Yes  No | 0  4 |
| Vision (follows hand)  Yes  No | 0  4 |
| Corneal reflex  Yes  No | 0  4 |
| Whisker movement  Yes  No | 0  4 |
| Hearing (turns to clapped hands)  Yes  No | 0  4 |
| Motor deficit (worst = 10 points)  Leg/tail movement  Normal  Stiff  Paralyzed | 0  5  10 |
| Sensory deficit (worst = 10 points)  Leg/tail (on pinching)  Yes  No | 0  10 |
| Coordination deficit (worst = 20 points)  Beam balance test  Walks the balance beam flawlessly and completes the walk within 6s  Walks the beam but is somewhat unsteady. Completes the walk within 6s  Walks the beam but is somewhat unsteady. May pause 1 or more times.  Takes > 6s to complete the walk  Walks the beam, but is very unsteady, almost falling off, may pause 1 or more times, and/or takes > 6s  Falls off the beam before completing the walk  Falls off the beam immediately | 0  2  4  6  8  10 |
| Prehensile Traction test |  |
| The rat hangs on for 5 s | 0 |
| The rat hangs on for 3–4 s | 5 |
| The rat hangs on for 0–2 s | 10 |
| General impression (worst = 10 points)  Normal except for the above  Abnormal except for the above (eg, hyperactivity and hypoactivity) | 0  10 |
| Total | 80 |
