# Supplementary material for: Sevoflurane Postconditioning-Induced Anti-Inflammation via Inhibition of the Toll-Like Receptor-4/Nuclear Factor Kappa B Pathway Contributes to Neuroprotection against Transient Global Cerebral Ischemia in Rats
Source: Int J Mol Sci. 2017 Nov 6;18(11):2347. doi: 10.3390/ijms18112347 (PMC5713316; doi:10.3390/ijms18112347)
Supplement: Supplementary file 1 [file ijms-18-02347-s001.zip › supplementary table 1.docx]

|  | | Group C  (*n* =10) | Group S  (*n* = 10) | Group R  (*n* = 10) | Group SR  (*n* = 10) | Group SH  (*n* = 5) |
| --- | --- | --- | --- | --- | --- | --- |
| pH | Baseline | 7.45 (7.44–7.45) | 7.44 (7.42–7.46) | 7.43 (7.41–7.45) | 7.43 (7.42–7.46) | 7.42 (7.40–7.47) |
|  | Ischemia | 7.36 (7.33–7.43) | 7.35 (7.33–7.37) ^§§^ | 7.37(7.35–7.38) ^§§^ | 7.38 (7.36–7.43) | 7.43 (7.41–7.44) |
|  | Reperfusion | 7.34 (7.31–7.38) ^§^ | 7.34 (7.30–7.38) ^§^ | 7.36 (7.31–7.40) ^§^ | 7.37 (7.32–7.38) ^§§^ | 7.41 (7.40–7.43) |
| PaCO_2_ (mmHg) | Baseline | 34.7 (32.6–36.6) | 34.4 (32.0–36.2) | 35.4 (32.3–38.0) | 36.7 (34.9–38.6) | 38.2 (32.9–38.5) |
|  | Ischemia | 35.1 (33.7–38.7) | 36.3 (33.0–38.0) | 34.7 (32.5–37.0) | 35.1 (30.3–38.9) | 37.6 (34.2–40.0) |
|  | Reperfusion | 37.4 (36.4–41.5) | 36.0 (33.8–40.0) | 36.7 (32.5–40.6) | 37.3 (35.9–40.4) | 37.9 (35.3–43.7) |
| PaO_2_ (mmHg) | Baseline | 245.1 (231.3–271.5) | 251.9 (234.1–265.8) | 246.3 (221.9–260.2) | 250.2 (225.9–266.2) | 270.3 (253.2–277.6) |
|  | Ischemia | 247.5 (231.6–254.5) | 243.5 (237.5–262.5) | 241.5 (228.1–245.8) | 247.6 (233.5–254.4) | 255.3 (241.2–366.9) |
|  | Reperfusion | 232.0 (220.3–246.1) | 225.4 (191.2–243.0) ^§^ | 228.1 (203.9–239.9) ^§§^ | 239.3 (189.3–242.4) | 251.6 (233.8–258.9) |
| Hb  (g/dL) | Baseline | 11.4 (11.0–11.8) | 11.0 (10.8–11.6) | 11.0 (10.6–11.5) | 10.8 (10.3–11.4) | 11.1 (10.9–11.6) |
|  | Ischemia | 10.4 (10.0–11.1) ^§^ | 10.5 (10.0–10.9) ^§§^ | 10.6 (10.2–10.9) ^§§^ | 10.6 (10.1–10.8) ^§^ | 11.0 (10.5–11.1) |
|  | Reperfusion | 10.6 (9.9–11.6) ^§^ | 10.8 (9.8–11.1) ^§^ | 10.5 (10.1–11.0) ^§^ | 10.7 (10.2–11.0) ^§^ | 11.0 (10.8–11.1) |
| Blood glucose  (mg/dL) | Baseline | 119.0 (111.3–150.8) | 138.0 (99.5–163.0) | 130.5 (92.8–157.3) | 133.0 (102.0–162.0) | 132.0 (106.0–169.5) |
|  | Ischemia | 122.5 (107.0–171.8) | 149.0 (118.0–164.0) | 124.0 (102.8–148.3) | 112.0 (96.8–122.0) | 121.0 (103.5–150.0) |
|  | Reperfusion | 125.0 (115.0–152.8) | 153.0 (128.5–176.0) | 135.5 (117.3–208.0) | 136.0 (111.0–169.0) | 141.0 (104.5–180.0) |
| MAP (mmHg) | Baseline | 81.0 (73.8–87.0) | 80.5 (76.3–87.8) | 82.5 (77.0–84.3) | 76.0 (73.0–81.0) | 80.0 (75.0–80.5) |
|  | Ischemia | 28.5 (27.8–29.3) ^§§^ | 28.0 (27.0–29.0) ^§§^ | 28.5 (26.0–29.0) ^§§^ | 29.0 (27.0–29.0) ^§§^ | 74.0 (72.0–80.5) |
|  | Reperfusion | 105.0 (93.8–115.0) ^**, ‡‡, §§^ | 62.5 (59.8–64.3) ^††,§§^ | 109.5 (97.8–120.3) ^‡‡, §§^ | 62.0 (60.0–64.0) ^§§^ | 74.0 (71.5–80.5) |
| Laser Doppler (PU) | Baseline | 88.8 (75.7–138.7) | 85.9 (78.8–93.5) | 105.6 (84.1–117.2) | 87.1 (78.9–111.8) | 94.2 (61.1–96.7) |
|  | Ischemia | 18.3 (11.3–29.3) ^§§^ | 16.7 (10.6–28.1) ^§§^ | 25.3 (19.8–41.2) ^§§^ | 19.2 (17.2–26.4) ^§§^ | 83.0 (66.8–106.4) |
|  | Reperfusion | 156.2 (130.3–190.2) ^**,‡, §§^ | 102.5 (90.9–128.0) ^†^ | 154.6 (113.5–203.2) ^‡, §§^ | 100.8 (82.8–136.5) | 80.6 (69.3–107.3) |

Table 1. Physiological variables during ischemia/reperfusion period. Values are presented as median with interquartile (Q1–Q3). All parameters are measured 10 min before ischemia (baseline), after 10 minutes of ischemia (ischemia), and after 30 minutes of reperfusion (reperfusion). Hb, hemoglobin; MAP, mean arterial pressure; PU, perfusion units; group C, control; group S, sevoflurane postconditioning; group R, resatorvid; group SR, sevoflurane postconditioning + resatorvid; group SH, sham.**: P < 0.01 vs. group S, ††: P < 0.01 vs. group R, ‡‡: P < 0.01 vs. group SR, §§: P < 0.01 vs. group SH. *: *p* < 0.05 vs. group S, †: *p* < 0.05 vs. group R, ‡: *p* < 0.05 vs. group SR, §: P < 0.05 vs. group SH.
