# Supplementary figures and images for: Sevoflurane Postconditioning-Induced Anti-Inflammation via Inhibition of the Toll-Like Receptor-4/Nuclear Factor Kappa B Pathway Contributes to Neuroprotection against Transient Global Cerebral Ischemia in Rats
Source: Int J Mol Sci. 2017 Nov 6;18(11):2347. doi: 10.3390/ijms18112347 (PMC5713316; doi:10.3390/ijms18112347)

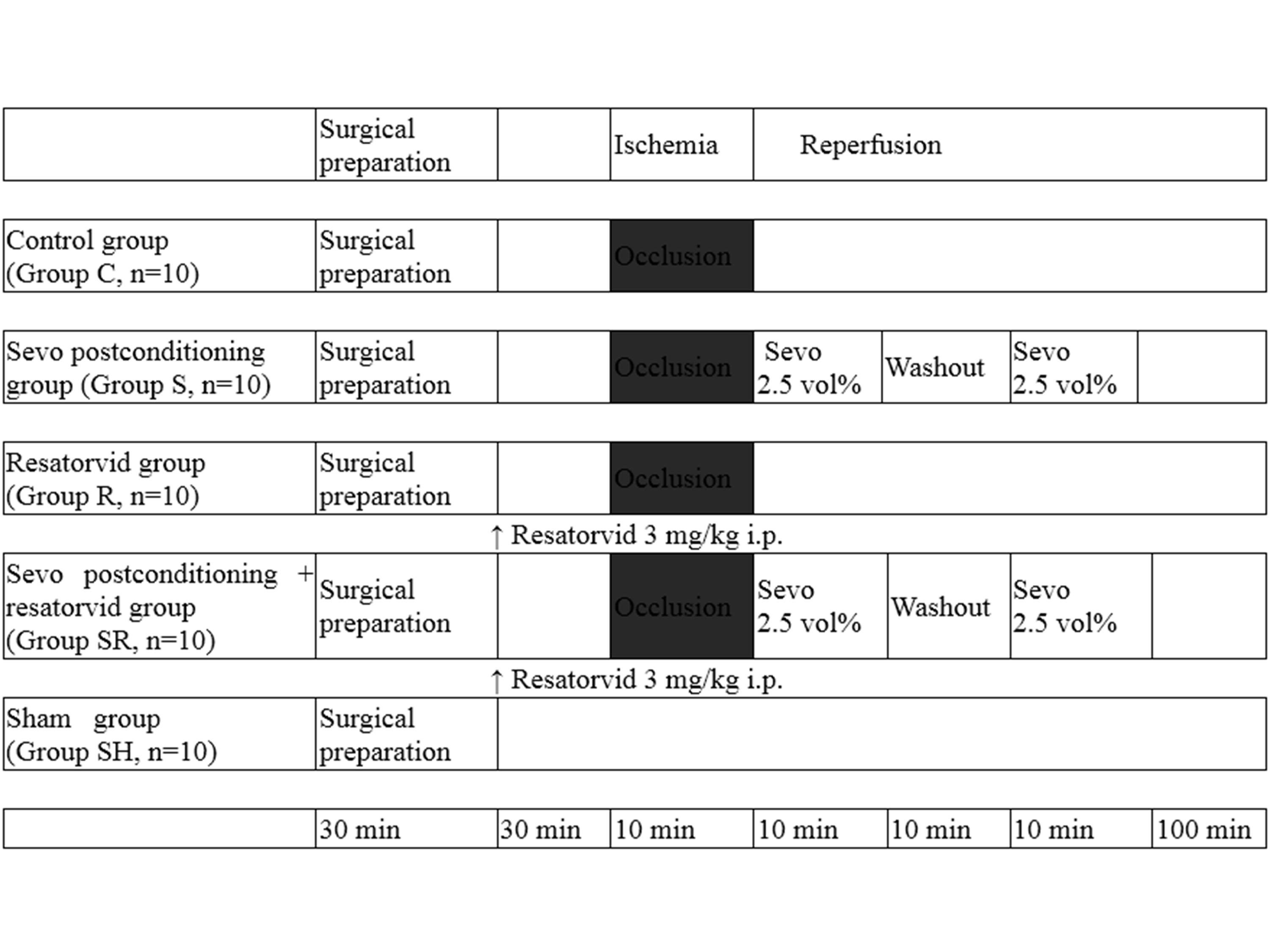

Supplement: Supplementary file 1 [file ijms-18-02347-s001.zip › supplementary figure 1.tif]
